# Parliament and the Profession

**Published:** 1915-11-27

**Authors:** 


### PARLIAMENT AND THE PROFESSION.

### Who Hoaxed the M.P.?

Apparently someone has been hoaxing the hon. member for the Haggerston Division. On November 16 he went to the House with a weird story about seven men of the R.A.M.C. "whose only crime is the exercise of their legal right to refuse inoculation," who had been punished by deduction in pay and by being put on extra fatigue duty, and had been repeatedly told that inoculation is compulsory. Mr. Tennant disposed of the matter by informing Mr. Chancellor that none of the statements had any foundation in fact.

### Soldiers in Hospital.

Mr. Chamberlain has promised to inquire into a matter which has been brought to his notice by Mr. R. McNeill, who has asked whether it is a fact that men on active service at Aden who had been compelled to go to hospital from the effects of the sun had 4½d. per day deducted from their pay while in hospital. If this be true, it seems very hard on the soldiers in question.

### Medical Service in the Highlands.

Replying to a question by Mr. Leicester Harmsworth on November 16, Mr. McKinnon Wood stated that a considerable proportion of the doctors in the area covered by the Highlands and Islands Medical Service Board had already entered into agreements with the Board for the improvement of medical attendance to the families of insured persons, crofters, farm servants, and others, and the Board was endeavouring to complete arrangements with all the practitioners before the end of the year.

### Administration of Military Hospitals.

On November 16 Mr. James Mason asked whether men of military age having experience of hospital administration work could be utilised in similar work for the War Office, instead of joining the combatant ranks. Mr. Tennant replied that the officers in charge of these hospitals must be qualified medical men. He added the cryptic statement, "I would suggest that the gentleman whom my hon. friend has in mind would do best to join the combatant ranks."

### Soldiers Suffering from Nerve Strain.

The Under-Secretary of State for War has already answered in the House of Commons something like sixty questions dealing with every aspect of the provisions made for the treatment of soldiers suffering from nerve strain. But on November 15 he was asked another question. Sir A. Markham wanted to know whether such soldiers, who have not been certified as insane, are being placed in annexes in county asylums. The effect of Mr. Tennant's reply was that it is the practice for soldiers who are ill from nerve shock to go to places which are within the precincts but outside the area of such institutions.

### Enlistment of Medical Students.

On November 18 Mr. Trevelyan asked the Under-Secretary for War whether he was aware that a special attempt was being made to enlist medical students of the first, second, and third years at Cambridge, and that if this policy was continued the entire supply for Cambridge was liable to be cut off for three or four years. He suggested that the Government should ask Lord Derby to cease this recruitment. Mr. Tennant replied that Lord Derby had seen the Presidents of the Colleges of Surgeons and Physicians, and that the present view was that such students should enlist or qualify for commissions.

### A Grievance from Ireland.

In the House of Commons on November 18 Mr. Byrne suggested that the Apothecaries' Hall of Ireland had recently been hindered in their work of qualifying surgeons for the Army by the action of the General Medical Council, "who have subjected them to a series of visitations, inspections, and inquisitions which have had the effect of frightening away a number of competent candidates who desired to get qualified and serve in the R.A.M.C." Mr. Tennant said that a complaint to this effect had been received from the Apothecaries' Hall, but the Army Council had no authority over a body such as the General Medical Council.

### The Cost of Domiciliary Treatment.

The high cost of the treatment of insured tuberculous persons formed the basis of several questions asked by Mr. Currie in the House of Commons on November 18 He asked whether the three cases receiving domiciliary treatment under the Insurance Committee for the county of Renfrew in 1913 cost on an average £327 11s. 5d. each; whether it had been made known that the estimated deficit of this committee's tuberculosis treatment fund for the current year amounts to £3,678; and whether the Treasury had been asked to pay one-half of this deficit and had agreed to do so. Mr. Roberts could not admit the validity of the estimate of the cost of the three cases in question, but the answers to the other two points were in the affirmative.

